# Cultural transmission results in convergence towards colour term universals

**DOI:** 10.1098/rspb.2012.3073

**Published:** 2013-05-07

**Authors:** Jing Xu, Mike Dowman, Thomas L. Griffiths

**Affiliations:** 1 Department of Neurology, Johns Hopkins University, 600 N Wolfe Street, Baltimore, MD 21287, USA; 2 ImageScope, 72 Melville Grove, Newcastle upon Tyne, UK; 3 Department of Psychology, University of California, 3210 Tolman Hall, MC 1650, Berkeley, CA 94720-1650, USA

**Keywords:** cultural evolution, colour term universals, world colour survey, iterated learning, Bayesian inference

## Abstract

As in biological evolution, multiple forces are involved in cultural evolution. One force is analogous to selection, and acts on differences in the fitness of aspects of culture by influencing who people choose to learn from. Another force is analogous to mutation, and influences how culture changes over time owing to errors in learning and the effects of cognitive biases. Which of these forces need to be appealed to in explaining any particular aspect of human cultures is an open question. We present a study that explores this question empirically, examining the role that the cognitive biases that influence cultural transmission might play in universals of colour naming. In a large-scale laboratory experiment, participants were shown labelled examples from novel artificial systems of colour terms and were asked to classify other colours on the basis of those examples. The responses of each participant were used to generate the examples seen by subsequent participants. By simulating cultural transmission in the laboratory, we were able to isolate a single evolutionary force—the effects of cognitive biases, analogous to mutation—and examine its consequences. Our results show that this process produces convergence towards systems of colour terms similar to those seen across human languages, providing support for the conclusion that the effects of cognitive biases, brought out through cultural transmission, can account for universals in colour naming.

## 1. Introduction

Cultural evolution involves two kinds of forces: those that affect *who* we choose to interact with, and those that affect *what* is transmitted through the interaction. These two kinds of forces have effects that are formally analogous to the effects of selection and mutation in biological evolution, and both contribute to the outcome of cultural evolution. Both kinds of forces can be expressed in mathematical models of cultural evolution [1–5], making it possible to ask what role these forces have played in producing the cultural practices of human societies. In particular, several researchers have recently emphasized that the fact that information is transmitted through human minds (rather than the process of copying with random mutations seen in biological transmission) creates the potential for cognitive biases to play a significant role in shaping the outcome of cultural evolution [6–8].

In this paper, we provide an empirical contribution which illustrates the powerful effect that cognitive biases can have on cultural evolution, using universals in systems of colour terms as a case study.^1^ Universals in systems of colour terms are well documented: languages with a small number of terms show characteristic patterns in which colours those terms label [11–13]. For example, when a language has three colour terms, one term usually denotes light colours, another dark colours and the third reddish colours [12,13]. We present the results of a laboratory simulation of cultural evolution in which we were able to observe the effect of mutation (cognitive biases) on systems of colour terms in the absence of selection pressures (preferences about who to learn from). Comparing the patterns that emerge purely as a result of the influence of human cognitive biases with documented patterns in human languages allows us to evaluate the role that the analogues of different evolutionary forces might play in accounting for cultural universals.

This paper is organized as follows. In §2, we present a mathematical framework that can be used to express the different forces that could be at work in cultural evolution, and to relate these forces to cultural universals. We then use this framework to outline our empirical strategy for evaluating the role of cultural transmission in producing patterns in colour term naming. The remaining sections of the paper present a large-scale experiment simulating the cultural transmission of colour terms in the laboratory, analyses of the results of this experiment and our conclusions about the potential roles played by different forces in producing cultural universals.

## 2. Mathematical models of cultural evolution

The impact of forces analogous to selection and mutation on the outcome of cultural evolution can be explored, using the standard model of evolutionary dynamics in large populations, the replicator dynamics [14]. In modelling biological evolution, the replicator dynamics describes how the proportion of the population of different types of organisms changes over time, assuming an infinite population. Let *x_i_* denote the proportion of a population that is of type *i* at a given moment *t*, and *q_ij_* the probability that an organism of type *i* is produced as the offspring of an organism of type *j* as a consequence of mutation. The population proportions then evolve as2.1
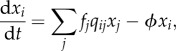
where *f_j_* is the *fitness* of holders of type *j*, in the sense of the mean number of offspring, 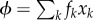 is the mean fitness and the second term on the right-hand side ensures that 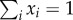.

The replicator dynamics can also be used to characterize cultural evolution [1,4]. Rather than a population of organisms of distinct biological types, we have a population of agents who entertain different cultural hypotheses, such as different variants of a language. The proportion of agents holding hypothesis *i* is then *x_i_*. Learning replaces biological transmission, and *q_ij_* becomes the probability that an agent ends up with hypothesis *i* after learning from an agent with hypothesis *j*. Errors resulting from cognitive biases that affect the hypotheses adopted through learning thus play a role in cultural evolution analogous to that of mutation in biological evolution. Finally, fitness is interpreted as influencing the probability with which an individual chooses to learn from an agent in the previous generation. If agents are selected with probability proportional to *f_j_*, then the same dynamics hold. Thus, factors that influence who learners choose to learn from play a role in cultural evolution that is analogous to selection in biological evolution.

Following this translation from biological to cultural evolution, equation (2.1) has been applied to the cultural evolution of languages, in the form of the ‘language dynamical equation’ explored by Nowak *et al*. [15,16]. In this paper, fitness is typically assumed to be a function of how well speakers of a particular language can communicate with the population at large, implementing a selection pressure for communication. If we instead assume that all speakers have equal fitness, *f_j_* = 1, then equation (2.1) simplifies to2.2
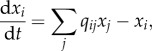
which is a linear dynamical system. This is a ‘neutral’ model, in which there are no selective forces favouring one language or hypothesis over another, making it possible to explore the effects of cultural transmission alone. A special case of this model was analysed by Komarova & Nowak [17].

**Figure 1.**
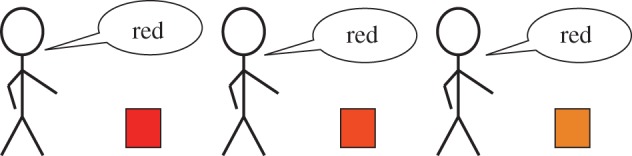
Cultural transmission by iterated learning. Systems of colour terms are transmitted by individuals who learn which words label which colours from examples and then produce labels to describe new colours. The labels produced by one individual become the examples from which the next individual learns, establishing a process of ‘iterated learning’.

The asymptotic behaviour of this linear dynamical system is straightforward to analyse: it converges to a stable equilibrium at the first eigenvector of the matrix **Q** = (*q_ij_*). This equilibrium is the same as that of another model used to explore cultural evolution, the iterated-learning model introduced by Kirby [18]. In iterated learning, a finite and discrete sequence of agents each learns from the previous agent in the sequence (figure 1). The result is a Markov chain, where the transition matrix of the Markov chain corresponds to the matrix **Q**. The stationary distribution of this Markov chain is the first eigenvector of **Q**. As the length of the chain increases, the probability that an agent selects a given hypothesis converges to this stationary distribution, matching the proportion of the population that would choose that hypothesis under the neutral form of the replicator dynamics [3].

In most work applying the replicator dynamics to cultural evolution, the mutation process represented by **Q** is assumed to be relatively unstructured. For example, Nowak *et al*. [15,16] assumed that languages are all equidistant from one another, meaning that agents had no biases towards learning particular languages. As a consequence, mutation does not favour any one language over any other, and the neutral model has relatively uniform equilibria. By contrast, work on iterated-learning models has explored accounts of cultural transmission that can incorporate perceptual and learning biases on the part of the agents. For example, Griffiths & Kalish [3] investigated the outcome of cultural transmission by Bayesian agents who select hypotheses by sampling from their posterior distribution. This approach makes it possible to quantify the biases of the agents, which are expressed in a prior distribution over hypotheses. Griffiths and Kalish showed that, in this case, the stationary distribution produced by iterated learning is just the prior distribution over hypotheses assumed by the agents. This result indicates that it is possible for forces analogous to mutation to have very strong effects on the outcome of cultural evolution, once possible biases on the part of the agents are taken into account.^2^

The relationship between the stationary distribution of iterated learning and the equilibrium of the neutral replicator dynamics creates an opportunity to empirically investigate the relationship between cognitive biases and cultural universals. Unlike the assumptions behind the replicator dynamics, which require interaction between individuals in order to implement selection, the assumptions behind iterated learning are easy to satisfy in a laboratory setting. We can thus use iterated learning as the basis for a laboratory experiment that reveals the equilibrium of cultural evolution in the absence of forces analogous to selection. If this equilibrium resembles the distribution of hypotheses seen across human societies for a particular domain (such as systems of colour terms), then cognitive biases may play a significant role in accounting for cultural universals in that domain.

## 3. Exploring the source of universals in colour naming

As a case study in evaluating the role that cognitive biases brought out through cultural transmission might play in accounting for cultural universals, we applied the iterated-learning approach to systems of colour terms. In addition to having been extensively studied, patterns in colour naming provide an interesting case study for teasing apart the evolutionary forces behind cultural universals, because both the structure of linguistic interaction (selection) and cognitive biases influencing transmission (mutation) have been proposed as potential explanations.

One group of researchers has argued that colour naming universals result from interaction—specifically the constraints induced by communicating with other people about colours. Steels & Belpaeme [19] showed that an interactive task in which agents seek to communicate effectively with one another about a set of colours resulted in the formation of a coordinated system of colour terms shared by the agents. Baronchelli *et al*. [20] showed that the partitions of the space of colours produced by these interactive language games shared some of the statistical properties of systems of colour terms seen in human societies.

Another group of researchers has focused on perceptual or learning biases of the kind that could influence cultural transmission. These researchers argue that human colour perception is an effective encoding of the physical structure of our environment [21,22], and that the systems of colour terms observed across human societies are good solutions to the problem of partitioning the space of colours given the properties of this perceptual system [23,24]. Finally, when English speakers are asked to partition colours, they produce partitions that are consistent with proposed cultural universals [25]. These results suggest that the nature of human colour vision and a preference for learning systems of colour terms that are good partitions of the resulting perceptual space might account for why these are the systems that are produced by cultural evolution.

Computer simulations of cultural evolution have been one of the main tools used to evaluate accounts of the patterns that appear in the systems of colour terms. Claims about the role of interaction have been justified through simulations of communication about colour between interacting agents [19,20]. Likewise, the effects of cultural transmission have been explored through computer simulations of iterated learning, in which a sequence of agents is constructed in which each agent learns a system of colour terms from examples provided by the previous agent, and then generates examples that are provided to the next agent in the sequence (figure 1). These simulations demonstrate that iterated learning with artificial agents can produce systems of colour terms that mirror universal patterns seen in human languages [26,27].

While computer simulations of cultural transmission of systems of colour terms suggest that the biases of individual learners can produce results consistent with observed universals, the conclusions drawn from these simulations are limited by the extent to which the artificial agents accurately mimic human perceptual and learning biases. In the remainder of the paper, we present a large-scale behavioural experiment with human participants who were designed to address this problem. In our experiment, human learners acquired and transmitted novel systems of colour terms by iterated learning, creating a laboratory simulation of the process of cultural transmission. We examined how these systems of colour terms change over time, comparing the resulting systems to those seen in human languages.

**Figure 2.**
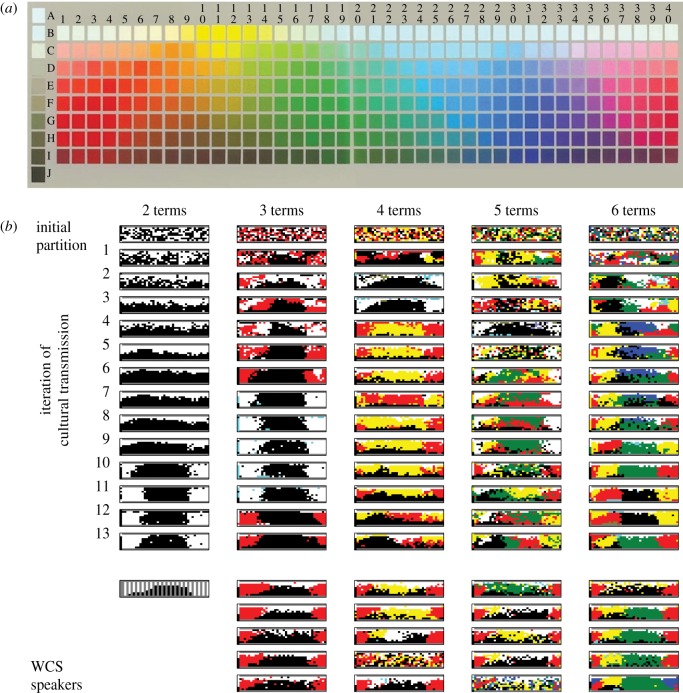
Simulating the cultural transmission of colour term systems. (*a*) The array of colours used in the world colour survey (WCS). Members of 110 non-industrial societies reported the terms that their languages used to label these colours [28–30]). (*b*) Examples of colour-term systems produced by simulating cultural transmission in the laboratory. Each column shows one chain of systems produced by participants learning novel colour terms from examples sampled from the system of labels assigned to colours by the previous participant. Transmission proceeds down the column, and different columns show chains for systems with two, three, four, five and six terms. The colour chips are arranged in the same order as in (*a*), so the position of a chip in the arrays corresponds to its colour. The colours signify which chips were labelled with the same colour word, and do not directly correspond to the colours denoted by the words (light blue is used to indicate a minority term, used for less than 5% of all chips). The first system in each column is a randomly generated initial partition. The last system in the first column is the Dani language [31], for which only aggregate data using a subset of the WCS array are available (the grey bars correspond to unlabelled chips). The last five systems in the remaining columns are data from individual speakers of the closest matching WCS language (determined by averaging variation of information (VI) values across iterations 4–13 of each chain).

The most definitive account of the regularities seen in systems of colour terms is provided by the world colour survey (WCS) [28–30]. In the WCS, a total of 330 colour chips, comprised 40 equally spaced Munsell hues at eight levels of lightness and achromatic chips at 10 levels of lightness (figure 2*a*), were presented to speakers of 110 different languages, all from non-industrial societies. Those speakers were asked to name each colour chip, and also to point out the most representative chip for each colour term. In our laboratory simulation of cultural transmission, participants were shown examples of colours labelled using a novel system of colour terms, and they then generalized these terms to other colours. The stimuli that were used were based on those from the WCS, with the goal of comparing the languages produced by our laboratory participants with those of the speakers of the WCS languages. In order to limit the influence of the English colour terms known by our experimental participants, we made it clear to participants that they were learning a novel artificial language. We also fixed the maximum number of terms in the artificial languages to between two and six terms, consistent with the number of terms seen in the majority of WCS languages, but significantly different from the 11 basic colour terms of English. This forced the participants to explore unfamiliar ways of partitioning the colour space.

Following the iterated-learning model (figure 1), we simulated the cultural transmission of languages by using the responses of one participant to train the next. Each participant was trained by being shown a set of randomly selected example colours, each paired with the word chosen to name it by the previous participant. The first participant in each chain was shown examples sampled from a computer-generated initial partition. After training, participants were asked to label all 330 WCS colours based on the examples they had seen. By examining the responses of our participants, we were able to determine the consequences of this simulated process of cultural transmission. We found that the systems of colour terms generated by our iterated-learning chains converged over time to become similar to those seen in non-industrial human societies, as documented by the WCS.

## 4. Methods

### (a) Participants

Participants were 399 members of the community at the University of California, Berkeley, receiving either course credit or approximately $10 per hour for taking part in the experiment. Participants had normal colour vision.

### (b) Stimuli

Each participant learned a system of colour terms from examples of colours and the terms that were associated with them, and they then generalized those terms to new colours. A total of 330 colours were used as stimuli, corresponding to the computer screen analogues of the 330 Munsell colour chips used in the WCS. Each term was a randomly allocated pseudo word consistent with the phonological system of English, and the words were varied randomly across participants. The colour stimuli were presented on an Apple iMac computer by a Java program.

Munsell values of the 330 colour chips were converted to values in CIE 1931 XYZ space and red, green, blue (RGB) space, using the GretagMacbeth Munsell conversion database v. 6.2. We then used those RGB values to present the 330 colour chips on the computer monitor. The monitor was calibrated, using a ColourVision Spyder2 colourimeter/colour calibrator on a regular basis. The accuracy of calibration was assessed, using a photometer (Minolta Chroma Meter CS-100, manufactured by Minolta Camera Co., Ltd., Osaka, Japan), confirming that the range of variation in the stimuli was small enough that no two colours from the stimulus set were confusable.

### (c) Procedure

We simulated a total of 30 iterated-learning chains, each with 13 ‘generations’ of learners. Each chain varied in the maximum number of terms that were allowed in the ‘language’ being transmitted, with two, three, four, five or six terms per language. The first learner in each chain received data generated from one of three types of initial partition of the WCS colour space: hue, lightness and random. The ‘hue’ and ‘lightness’ partitions were approximately equal vertical and horizontal partitions of the colour space into the relevant number of categories. (In the ‘hue’ division, the achromatic chips were grouped with a randomly chosen hue partition.) The ‘random’ partitions were a truly random partition of the colour space, with an equal number of instances of each term, and were generated uniquely for each chain. These three kinds of initial partition were used as a means of checking the convergence of iterated learning: by starting the chains with very different systems of colour terms, we could easily establish when the influence of the initial partition had disappeared. The following generations of learners all received data generated from the responses of the previous generation, as detailed below. We ran a total of 20 random chains, four for each number of terms, and five hue and five lightness chains, one for each number of terms.

Each participant was trained on the system of colour terms by being shown a set of colours together with the corresponding terms. The total number of observed colours was six times the number of terms in the language. These chips were chosen at random from the 330 colours making up the full array, and were labelled according to either the initial partition (for the first learner) or the responses of the previous learner (for subsequent participants). In order to reduce the memory demands of the task, these training examples remained on the screen while the participant went on to label all 330 colours from the WCS array. On every trial, the participant was presented with a colour, and was then asked to select one of the terms to label that colour. No feedback was given during this phase of the experiment. The responses of each participant thus produced a partition of the set of 330 colours, and this partition was used to generate the labels given to colours for the next learner in the chain.

To rule out the hypothesis that our iterated-learning chains simply converged on English, we also conducted another experiment in which nine English-speaking participants were asked to label the 330 WCS colours with one of the six English colour terms (*black*, *white*, *red*, *green*, *yellow* and *blue*). The experiment was conducted in exactly the same environment as the iterated-learning experiment. On each trial, the participant was presented with a colour and was asked to select one of the English terms to classify the colour.

## 5. Results

Figure 2*b* shows one set of chains initialized with random partitions, with the number of terms varying from two to six. Through this simple visualization of the data, we can see that each chain started from an unnatural colour-term system, and that transmission along the chains resulted in a rapid restructuring towards a more regular form. Our primary analysis compared the systems of colour terms produced by our participants with those in the WCS, and showed that the two became more similar as the number of iterations of transmission increased. Subsequent analyses were conducted to address two possible concerns about the source of this increase in similarity. The analyses we present in this section focus on the chains initialized with random partitions—the use of the hue and lightness partitions to assess convergence is discussed in the electronic supplementary material.

### (a) Comparison with the world colour survey

Analysing the results of our experiment presented a challenge: how could we evaluate whether two systems of colour terms were similar? Various methods have been proposed for solving this problem. For example, Kay & Regier [32] converted the colour chips from Munsell space to CIE L*a*b* space so they could compute the centroid for each colour term. Centroid distances could then be used to compare clusterings. However, just using centroid measurements might have discarded important information about the variance of a cluster, and about the locations of boundaries. This method is also dependent on the psychological validity of the CIE L*a*b* representation of colours, which is disputable [26].

Because our participants’ responses consisted of partitions of the same set of colours as those used in the WCS, we compared the Munsell arrays directly, without referring to another colour space. Our technique used an information-theoretic measure known as variation of information (VI), which is able to quantify the similarity of alternative clusterings of a set of items [33] (we consider alternative measures in the electronic supplementary material). Given two clusterings *C* and 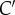, the VI is5.1
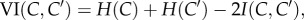
where *H*(*C*) is the *entropy* of *C*,5.2
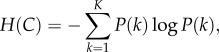
where *k* ranges over the cluster labels and *P*(*k*) is the probability of an item being assigned to each cluster, and 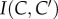 is the *mutual information* between the two clusterings:5.3
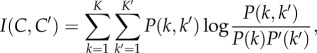
where 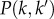 is the probability an item belongs to cluster *k* in clustering *C* and to 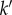 in clustering 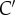.

We used VI to measure the similarity between the systems of colour terms produced by our participants and those in the WCS. The VI value for two systems of colour terms was calculated by comparing the relative frequencies of the terms in the two systems, as well as the extent to which they partitioned the colour space in the same way. A high VI value reflects a larger difference between two colour term systems, whereas a small VI value indicates that the two systems are more similar.

At the bottom of each chain in figure 2*b*, we show five randomly selected speakers from the closest matching system from the WCS according to the VI measure. Because there was no two-term language in WCS, we compared the two-term case with the Dani language [31]. To provide a quantitative analysis of the similarity between the systems of colour terms produced by our participants and those observed in the WCS data, we calculated the mean distance between these two sets of partitions. We first calculated the VI between each system produced by our participants and each system produced by the speakers of each language in the WCS, and then averaged across all speakers within each WCS language, then across all languages. A similar analysis restricted to languages with the same number of terms is presented in the electronic supplementary material.

**Figure 3.**
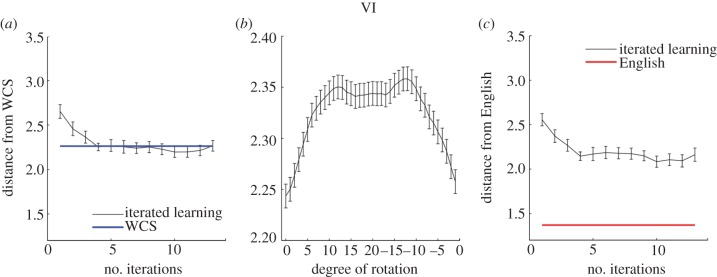
Comparing systems of colour terms produced by simulating cultural transmission in the laboratory with those from non-industrial societies, as represented by the world colour survey (WCS) and English colour systems. The distance between systems is assessed using variation of information (VI), an information-theoretic measure of the difference between two partitions of the same set of elements [33]. (*a*) The mean VI between systems produced by English-speaking participants and those seen in the WCS decreases as the number of iterations of cultural transmission increases. The VI converges to a level that is not statistically significantly different from the mean VI between languages in the WCS. (*b*) Taking the final systems produced by our laboratory participants, rotation along the hue dimension of the WCS array results in a worse fit to the WCS, confirming that there are significant similarities between the way the languages emerging in our experiments, and those spoken in non-industrial societies, categorize colours in terms of the hue dimension in the WCS. The horizontal axis indicates the number of steps the chips were rotated to the right along the hue dimension of the array shown in figure 1. (*c*) The mean VI between systems produced in the iterated-learning experiment and those produced by participants applying English colour terms. The VI between systems in iterated-learning chains and English systems did not reduce to the same level as the mean VI between systems produced by English speakers. (Online version in colour.)

Figure 3*a* shows the results of our comparison between the systems of colour terms produced by our participants and the WCS. The distance between chains with random initial partitions and the WCS clearly decreases across iterations. A paired *t*-test on the VI values for the initial and final systems in the 20 random chains showed a statistically significant difference (*t*_19_ = 5.83, *p* < 0.001). The remaining question is how close our data are to the WCS data: what counts as a low VI score? To address this question, we computed the VI between all pairs of languages in the WCS. The average pairwise VI is shown in figure 3*a*. This average is extremely close to the mean VI seen in our random chains once they converge. The difference between the VI scores for the systems produced by the final participants in each of our random chains and the VI scores for individual speakers of languages from the WCS was not statistically significant by a two-sample *t*-test (*t*_128_ = 0.09, *p* = 0.93), and in fact no statistically significant differences were observed from the fourth iteration onwards.

### (b) Rotation analysis

One potential objection to the conclusion that our chains converged to a distribution over partitions similar to that in the WCS could be that the reduction in VI may merely be a result of giving the same label to neighbouring colours, producing a more coherent classification of the colour space. As the systems of colour terms in the random chains move towards more regular forms, the VI scores will go down naturally, regardless of whether the actual partition of terms reflects the structure of the WCS or not. To further test the consistency between the results of our experiment and the WCS data, we compared the degree of match of each system with the WCS when it was rotated in the hue dimension (the horizontal dimension for the array shown in figure 2) by varying amounts. If the systems produced by our participants provide a non-trivial match to the WCS, we would expect that the more a partition was rotated out of position the worse the resulting match would be. If regularity alone is responsible for the match, then rotation should have no effect. A similar procedure was previously used by Regier *et al*. [24] to provide evidence for universals in colour naming. Figure 3*b* shows the mean VI values for the partitions generated by the final participants in each of our random chains, when rotated from 0 to 20 steps in the hue dimension. Paired *t*-tests on VI values for no-rotation versus maximum-rotation (*t*_19_ =−5.95, *p* < 0.001), no-rotation versus quarter-rotation (*t*_19_ =−3.45, *p* < 0.01) and no-rotation versus three-quarter-rotation (*t*_19_ =−4.51, *p* < 0.001) all showed statistically significant differences, indicating that the partitions produced by our participants fit the WCS data significantly better than the rotated systems. This analysis thus confirmed that the iterated-learning chains did converge to systems reflecting the patterns in the assignment of terms to colours of different hues that is evident in the WCS data.

### (c) Comparison with English

Another concern might be that the systems produced by our participants are converging to the English colour system, because all of our participants were speakers of English. The question is then how close our iterated-learning chains are to English colour systems. To compare our results with English, we conducted another experiment in the same laboratory environment, and asked English-speaking participants to label each of the WCS colours with one of six basic English colour terms, as mentioned in §4. We then computed the VI values between systems produced by the iterated-learning chains with random initial partitions and those in the English experiment in the same way as we did for the WCS data. Figure 3*c* shows the results of this comparison. Although the distance between iterated-learning chains and English systems decreases across iterations, it was still quite far from the average pairwise VI values among English systems: a two-samples *t*-test showed a statistically significant difference (*t*_27_ = 6.98, *p* < 0.001) between the VI scores produced by the final participants in each chain and those for the English systems (and likewise for all other iterations). This indicates that the classifications produced by different participants using English terms were more similar to each other than they were to the results of our iterated-learning experiment, suggesting that participants did not simply apply English colour categories when classifying colours in the iterated-learning chains.

## 6. Conclusion

Our experiment shows that systems of colour terms transmitted in the laboratory converge towards forms consistent with the WCS. Using VI as a measure of the difference between systems of colour terms generated in our experiment and the WCS data, we showed that the VI for systems generated by iterated learning rapidly decreases as the systems move from unnatural random partitions to more regular forms. Our rotation analysis confirmed that this reduction of VI cannot be explained as simply a result of the emergence of more regularity, but reflects the adoption of a form consistent with the WCS data. These results provide support for the idea that the learning and perceptual biases of human learners, brought out through cultural transmission, can account for the regularities observed in systems of colour terms across human languages (consistent with the explanations provided by earlier studies [21–25]).

The participants in our experiment were all English speakers, which makes it difficult to separate general cognitive biases shared by all human language learners from the effects of either prior exposure to English or prior exposure to language in general. We took steps to reduce the influence of English colour terms on our participants’ responses, such as forcing participants to use a restricted number of terms, and we conducted a control experiment showing that the final solutions produced by our participants were less consistent with one another than those produced by participants explicitly using English terms. However, to truly control for the effects of prior language exposure, it would be necessary to conduct an experiment in which participants were speakers of a range of languages, sampling these participants in a way that captures the variation in human languages. Because this requires using speakers from non-industrial societies, collecting such data would be a challenge on the same scale as the WCS itself. While finding that convergence towards the WCS occurs with English speakers provides evidence for a significant influence of cognitive biases, future work using a more diverse set of participants would be needed to strengthen this conclusion.

Returning to the broader issue of the factors that affect cultural evolution, this case study suggests that there may be cultural universals for which much of the explanatory weight falls on cognitive biases brought out through cultural transmission (consistent with earlier studies [6–8]). This does not rule out a role for forces analogous to selection, which are undoubtedly important in ensuring that members of a given society are consistent in their language and cultural practices and can have important effects on the accumulation of innovations [1]. Understanding how these different forces interact to produce cultural universals is an important challenge for research in cultural evolution, and we anticipate that combining mathematical analysis with laboratory simulations will be an important step towards addressing that challenge.

## Endnotes

1 Consistent with widespread practice in linguistics, our use of ‘universal’ includes observations about how frequently particular features are observed in languages from throughout the world. These ‘statistical universals’ contrast with absolute universals: properties that are common to all languages [9,10].

2 This analysis also provides a way to understand the *variation* that appears in languages as well as their regularities. The fact that the stationary distribution is the prior implies that, at equilibrium, the probability that an agent adopts a particular hypothesis is the prior probability of that hypothesis. Convergence to this distribution, rather than a single hypothesis, means that we should expect to see variation in languages consistent with what would be seen in multiple draws from the prior distribution. Every language can be different, particularly if this prior distribution assigns probability to a wide range of languages.
